# Accelerated theta-burst repetitive transcranial magnetic stimulation for depression in South Africa

**DOI:** 10.4102/sajpsychiatry.v25i0.1346

**Published:** 2019-11-27

**Authors:** Erine Bröcker, Leigh van den Heuvel, Soraya Seedat

**Affiliations:** 1 Department of Psychiatry, Faculty of Medical Health Sciences, Stellenbosch University, Cape Town, South Africa

**Keywords:** theta-burst stimulation, TBS, Depression, accelerated repetitive transcranial magnetic stimulation, bipolar depression, BP, treatment resistant depression

## Abstract

This case series documents local experience using accelerated theta-burst repetitive transcranial magnetic stimulation (rTMS) as a supplementary treatment for depression in both major depressive disorder (MDD) and bipolar disorder (BD). Nine consenting patients (MDD = 7; BD = 2) each received 20 accelerated theta-burst sessions over 8 days. Improvement was monitored using the Centre for Epidemiological Studies Depression (CES-D) Scale and the Clinical Global Impression (CGI) Scale at baseline, at day 5 and at day 8 of rTMS treatment. We performed a Wilcoxon matched-pairs signed-rank test to determine whether there was a difference in scores from baseline to post-treatment. The CES-D scores decreased significantly from baseline to post-treatment (*Z* = −2.547, *p* = 0.011) with five patients experiencing at least a 50% symptom reduction on the CES-D. CGI severity scores also decreased significantly between baseline and post-treatment (*Z* = −2.547, *p* = 0.011). The most commonly reported adverse effect was mild headaches, lasting a few hours during and after rTMS treatment. The findings suggest that the accelerated theta-burst rTMS protocol for depression is well tolerated with most patients also experiencing symptomatic improvement by day 8.

## Introduction

Transcranial magnetic stimulation (TMS) is a non-invasive brain stimulation technique that is based on the principle of magnetic induction.^1,2,3^ As a research tool, TMS has been widely used to investigate motor conduction time, motor function, and pathology in a variety of brain disorders.^2,4^ Transcranial magnetic stimulation generates an electromagnetic field of sufficient magnitude that ultimately results in altered neuronal activity in targeted focal cortical tissue.^5^ When pulses are applied repetitively, a modality referred to as repetitive transcranial magnetic stimulation (rTMS), TMS can modulate cortical activity beyond the stimulation period and thus has the potential to be used therapeutically. In resent years the research and clinical use of rTMS in the treatment of neuropsychiatric disorders has been expanding since.^1,2,4,5^

Further therapeutic application of TMS includes the development of theta-burst stimulation (TBS), where pulses are delivered in a pattern simulating the brain’s cortical theta rhythm.^6,7^ During TBS, three magnetic pulses are delivered 20 ms apart and repeated every 200 ms, generating a 5 Hz theta rhythm with more robust and long-term cortical excitability changes.^6^ Similar to other therapeutic applications, the exact mechanism of action of TBS remains to be elucidated. Studies suggest that the effects of TBS may be related to the modulation of γ-aminobutyric acid (GABA) interneuron inhibitory transmission affecting synaptic plasticity through processes such as long-term depression (LTD) and long-term potentiation (LTP).^8^ TBS appears to have certain benefits compared to traditional rTMS modalities, including shorter treatment duration and lower stimulation intensity. Studies suggest that the therapeutic effects are generally sustained.^6,7,8,9^ Additionally, research supports the safety of TBS application and the absence of clinically significant adverse effects.^7,9^

Studies on the application of accelerated intermittent TBS (iTBS) for major depression have yielded promising results.^10,11^ When rTMS is applied in the treatment of depression (both unipolar and bipolar), it is most commonly delivered to the dorsolateral prefrontal cortex (DLPFC).^10,11,12,13^ The DLPFC is involved in complex cognitive and behavioural functions and, of significance to depression, plays an essential role in the reappraisal or suppression of negative affect.^12^ Functional neuroimaging studies implicate the DLPFC in the regulation of negative emotions in depression.^12^ Because of the functional asymmetry observed in depressed patients, rTMS protocols administered in depression generally involve excitatory protocols to the left DLPFC and inhibitory protocols to the right DLPFC.^12,13^

Accelerated protocols involve multiple sessions of TBS per day as compared to the standard single session per day. A recent randomised double-blind sham-controlled crossover study focussed on increasing the effectiveness while shortening the duration of TBS in patients with treatment-resistant depression demonstrated that 4 days of TBS treatment over the DLPFC resulted in significant clinical improvement extending up to 2 weeks post-TBS treatment.^11^ Considering the practical advantages, in terms of decreased treatment duration and possibly faster treatment effects, accelerated protocols can potentially improve the clinical utility of rTMS.

Internationally, research on the clinical applications of rTMS is expanding; however, until recently rTMS treatment was largely unavailable in South Africa. The Department of Psychiatry at Stellenbosch University now runs an rTMS clinical and research service available to patients from both the private and public sectors (see Box 1). The following illustrative case series aims to document the effectiveness of the accelerated theta-burst rTMS protocol in patients with either unipolar or bipolar depression in South Africa. The case series highlights the short-term effectiveness, practicality and safety of an accelerated TBS protocol. It provides information on patient presentation, protocol selection and treatment outcomes and adds to the limited body of research on rTMS in South Africa.

BOX 1 Overview of the repetitive transcranial magnetic stimulation treatment service provided.

The rTMS service aims to individualise rTMS treatment based on the best available research, taking into consideration practicality and affordability for the local patient demographic. Patients with mental disorders are often faced with challenges such as cost, time away from work, transport and the need for more immediate and rapid therapeutic effects. One approach to address these challenges is to use the described accelerated theta-burst protocol version. At our rTMS clinic, the first step entails receiving a referral from the treating physician. The team then evaluates the suitability of the patient for rTMS treatment and identifies appropriate rTMS evidenced-based protocols for the identified condition. Suitable protocols, supporting literature, cost and practicality of protocols are provided to both the treating physician and patient to facilitate the decision-making process. Once treatment is initiated, individual progress is monitored for symptom change and global outcome measures, and feedback is provided to the referring physician post-treatment.

rTMS, repetitive transcranial magnetic stimulation.

## Methods

### Subjects

Nine patients with diagnosed and documented treatment-resistant depression, both unipolar and bipolar disorders (BDs), who were referred to the rTMS service were included in this case series (see Table 1). All patients signed informed consent and ethics approval was obtained from the Health Research Ethics Committee at the Faculty of Medicine and Health Sciences, Stellenbosch University (C18/05/01). None of the patients had documented comorbid disorders and none had any contraindications to rTMS treatment. All patients continued on their current treatment regimes, with rTMS added as an adjunctive treatment.

**TABLE 1:** Subjects and clinical data.

Patient (Pt)	Age	Gender	Diagnosis	Treatment	CES-D T 1†	CES-D T 2‡	CES-D T 3§	CGI T 1	CGI T 2	CGI T 3
Pt 1	57	F	MDD	Venlafaxine 300 mg Molipaxin 100 mg Sulpride 250 mg Rivotril 1.5 mg	35	33	19	6	5	4
Pt 2	34	F	MDD	Quetiapine 475 mg Lamotrigine 200 mg Mianserin 90 mg Lithium 750 mg	52	50	32	5	4	3
Pt 3	49	F	MDD	Nevirapine Tenofovir Lamivudine Lamotrigine 150 mg Mianserin 30 mg	40	22	42	4	4	4
Pt 4	33	F	BD	Abilify 5 mg Lorien 40 mg Lithium 750 mg Eltroxin 50 mg Epitec 100 mg Vitamin D	50	16	23	4	2	2
Pt 5	34	F	MDD	Selegline 200 mg Wellbutrin 300 mg Clonazepam 0.5 mg	38	29	6	5	4	3
Pt 6	37	F	MDD	Venlafaxine 300 mg Lamotrigine 200 mg Haloperidol 1.5 mg Proselgine 25 mg	58	19	16	5	5	4
Pt 7	43	F	MDD	Bupropion 450 mg Venlafaxine 475 mg Lithium 750 mg	27	16	16	4	3	3
Pt 8	32	M	BD	Epitel 250 mg Venlor 75 mg Wellbutrin 450 mg Seroquel 200 mg Urbanol 5 mg Stilnox 12.5 mg	37	14	17	4	3	3
Pt 9	47	M	MDD	Venlafaxine 375 mg Propanol 20 mg Lamotrigine 250 mg	20	5	6	3	2	3

CES-D, Centre for Epidemiological Studies Depression Scale; CGI-S, Clinical Global Impression Scale; MDD, major depressive disorder; BD, bipolar disorder; F, female; M, male.

† , T1 = Time 1 (Baseline);

‡ , T2 = Time 2 (Day 5 Post 11 rTMS accelerated sessions);

§ , T3 = Time 3 (Day 8 Post 20 rTMS accelerated sessions).

### Treatment

All patients received the accelerated iTBS protocol for depression which consists of three magnetic pulses delivered 20 ms apart and repeatedly delivered every 200 ms resulting in a 5 Hz theta rhythm over the left DLPFC area. Intensity was set to 80% motor threshold (MT), and 1782 pulses were delivered per session each lasting roughly 13 min. Treatment was administered with a MAG & More PowerMAG 100 rTMS stimulator and a double-coil PMD70-pCool figure-8 coil. The coil was placed at 45° angle targeting the left DLPFC, 5.5 cm parasagittal anterior to the motor hotspot.

Treatment sessions were arranged according to the availability of both the TMS administrator and patient and visits thus occurred at different times of the day over a total of 8 days (spread over 2 weeks). The first visit, lasting approximately an hour, entailed identification of anatomical landmarks as per standard rTMS procedure, MT determination using an electromyography (EMG) device and administration of a single rTMS theta-burst session. On days 2 and 3, patients received two rTMS theta-burst sessions with a 20-min safety break between sessions, resulting in visits of roughly an hour. Days 4 to 8 were characterised by three rTMS theta-burst sessions per day, again with a 20-min safety break between sessions, resulting in visits lasting roughly 90 min. The 20-min break in between rTMS sessions from days 2 to 8 was incorporated in accordance with rTMS protocol safety procedures.^10^

We designed a protocol that increased the number of sessions per day gradually to assess individual safety and enhance tolerability. Each patient collectively received 20 rTMS theta-burst sessions. Improvement was monitored at baseline and at day 5 and day 8 of treatment using the Centre for Epidemiological Studies Depression (CES-D) Scale and the Clinical Global Impression (CGI-S) Scale. Patients were asked daily about adverse events.

### Statistical analysis

A non-parametric Wilcoxon matched-pairs signed-rank test was used to determine whether there was a significant difference in scores between each time point (Time 1 = baseline, Time 2 = Day 5, and Time 3 = Day 8). Responders were defined as those with at least a 50% reduction in CES-D scores between baseline and time 3.

### Ethical considerations

All patients signed informed consent and ethics approval was obtained from the Health Research Ethics Committee at the Faculty of Medicine and Health Sciences, Stellenbosch University (C18/05/01).

## Results

A total of nine patients were included in this case series (seven females and two males, mean age = 40.67, standard deviation [s.d.] = 8.79). Seven of the nine patients had major depressive disorder, while the remainder was referred with BD (current episode of major depression). There was a decrease in depression scores as measured by the CES-D from Time 1 (*M* = 39.67; s.d. = 12.11) to Time 2 (*M* = 22.67; s.d. = 13.15) and from Time 2 to Time 3 (*M* = 19.89; s.d. = 11.85). The reduction in CES-D scores from Time 1 to Time 2 (*Z* = −2.668, *p* = 0.008) and Time 1 to Time 3 (*Z* = −2.547, *p* = 0.011) but not Time 2 to Time 3 (*Z* = −1.342, *p* = 0.180) was significant. Five of the nine participants were responders with an average reduction in CES-D score of 50.1%. The mean CGI severity score was 4.44 (s.d. = 0.88) at Time 1, 2.11 (s.d. = 0.60) at Time 2, and 3.11 (s.d. = 1.66) at Time 3. Again the improvement in CGI-S score from Time 1 to Time 2 (*Z* = −2.530, *p* = 0.011) and from Time 1 to Time 3 (*Z* = −2.547, *p* = 0.011) but not from Time 2 to Time 3 (*Z* = −1.342, *p* = 0.180) was significant. The most commonly reported adverse effect was mild headaches during and post-rTMS treatment lasting a few hours.

## Discussion

This was a small case series without a control arm; however, according to our knowledge this is the first documented report on the use of rTMS in South Africa. The results are promising as all patients, excluding one, experienced symptomatic improvement at termination of treatment.

Similar to other studies, patients included in this series received rTMS as an adjunctive treatment to their established medication treatment regimen.^14,15,16^ Furthermore, our results indicate that the largest clinical difference occurred between baseline and day 5, providing a supported rationale for recommending a minimum of 5 days of treatment (11 accelerated rTMS sessions) in our setting. Our results are consistent with other studies with regard to the efficacy of theta-burst rTMS treatment for treatment-resistant depression.^10,11,17^

The accelerated protocol also results in decreased overall treatment durations, which translated into decreased treatment cost and a decrease in time away from work for patients. Accelerated TBS protocols may offer benefits in terms of a faster response and a shorter duration of treatment, thus enhancing cost-effectiveness and practicality. Lastly, patients involved in this documented series experienced few side effects, with the most commonly reported one being mild headache. Thus, the accelerated theta-burst protocol appears to be well tolerated by patients.^10^ Although TBS appears to have similar safety and tolerability to traditional rTMS protocols, theoretically it does carry an increased risk because of higher frequency burst stimulation. Further research is needed to elucidate the mechanisms underlying the mechanistic effects of TBS and to establish ideal protocol parameters.

The results reported here are from a case series and further well-designed randomised controlled trials are required to demonstrate efficacy. However, the patients referred to the rTMS service are largely treatment resistant or treatment refractory, and thus, the case series illustrates the utility of an accelerated TBS protocol in real-world clinical cases.
